# Radiotherapy dosimetry and radiotherapy related complications of immediate implant-based reconstruction after breast cancer surgery

**DOI:** 10.3389/fonc.2023.1207896

**Published:** 2023-10-10

**Authors:** Yu Zhang, Fuxiu Ye, Yun Teng, Jin Zheng, Chunlu Li, Ruilan Ma, Haichen Zhang

**Affiliations:** Department of Radiation Oncology, The Second Affiliated Hospital of Dalian Medical University, Dalian, China

**Keywords:** modified radical mastectomy, breast reconstruction, intensity modulated radiation therapy, adverse reaction to radiotherapy, quality of life

## Abstract

**Backgrounds:**

The impact of immediate implant-based breast reconstruction (IBBR) on the delivery of radiotherapy plans remains controversial. This study aimed to compare the differences in radiotherapy dosimetry, complications of radiotherapy, and quality of life in patients who underwent modified radical mastectomy combined with or without IBBR.

**Methods:**

We retrospectively collected 104 patients with breast cancer who underwent intensity-modulated radiation therapy after modified radical mastectomy with IBBR (n =46) or not (n =58) from January 2017 to December 2021. The dosimetric differences in radiotherapy of planning target volume (PTV) and organs at risk and the differences in complications of radiotherapy between the two groups were compared. We also applied the functional assessment of cancer therapy-breast cancer (FACT-B) score to compare the difference in quality of life. The chi-square test and independent samples t-test were used to analyze the above data.

**Results:**

IBBR group was associated with higher PTV volumes, PTV D98, V95, and lower PTV Dmean, D2 compared with the non-reconstruction group (P<0.05). IBBR group also had lower radiotherapy dosimetric parameters in the ipsilateral lung and the heart of left breast cancer patients. The differences in the rates of radiation pneumonia (RP) and radiation dermatitis (RD) between the two groups were not statistically significant (P > 0.05). Moreover, FACT-B scores at 6 months after radiotherapy in patients with IBBR were higher than those without reconstruction (P < 0.05).

**Conclusion:**

Patients with IBBR achieved better radiation dosimetry distribution and higher quality of life without more complications of radiotherapy.

## 1 Introduction

According to the 2020 global cancer data released by the International Agency for Research on Cancer (IARC), breast cancer has become the cancer with the highest incidence rate around the world (1). Surgery is irreplaceable in improving the local control rate of breast cancer patients. The surgical approach has always been in evolution (2). Randomized controlled trials have proved that breast-conserving therapy (BCT) combined with radiotherapy is not statistically different from modified radical mastectomy (MRM) in terms of local control rates and long-term survival (3). And the cosmetic result of BCT is better. However, not all patients are suitable for BCT and MRM is still popular in China. As to the positive psychosocial benefits of breast reconstruction, more surgeons and patients perform reconstructions (4, 5). Compared with delayed reconstruction and delayed-immediate reconstruction, immediate breast reconstruction (IBR) has advantages in avoiding secondary surgery and breast loss (6, 7). Studies have also shown that immediate reconstruction does not affect survival or recurrence rates in breast cancer patients (8–10). In addition, although implant-based reconstruction has some complications, including infection, skin necrosis, implant rupture, capsular contracture, and even requires reoperation, it has become the most common breast reconstruction method in recent years because of its shorter surgery, hospitalization and recovery time, and no scar at the donor site (6, 7, 11). Agarwal et al. used the SEER database to count the IBR rate from 2000–2010 in patients requiring radiation, and the rate of IBR increased from 13.6% to 25.1%. The percentage of patients receiving implant-based reconstruction increased from 27% to 52% with a decrease in autologous reconstruction from 56% to 32% (12).

Postmastectomy radiation therapy (PMRT) can both reduce the risk of local recurrence and improve the survival rate of patients with high risk factors, such as the maximum diameter of the primary tumor ≥5 cm and positive axillary lymph nodes (13–15). However, the impact of implant-based breast reconstruction (IBBR) on the delivery of PMRT remains controversial. Besides, there are insufficient data in the literature about the effects of IBBR on the acute toxicity of radiotherapy and quality of life in patients undergoing PMRT. So in the context of the popularization of intensity-modulated radiotherapy (IMRT), we collected 104 patients who received IMRT after MRM and analyzed the effects of IBBR on radiotherapy dosimetry and radiotherapy related complications.

## 2 Materials and methods

**Figure 1:**
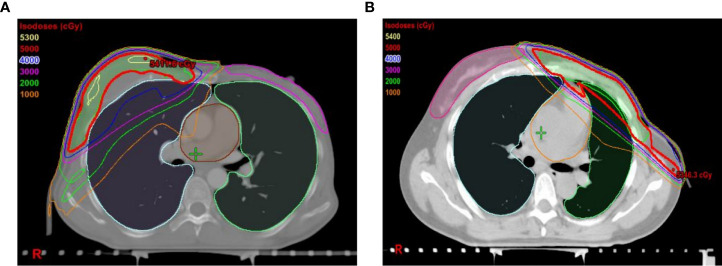
Images of the intensity modulated radiation therapy plan of patients after modified radical mastectomy. **(A)** Patients undergoing modified radical mastectomy combined with immediate implant-based breast reconstruction. **(B)** Patients undergoing simple modified radical mastectomy. Isodose lines (IDLs) are generated to describe where the radiation dose is distributed. Levels in cGy: red = 5000, blue = 4000, purple = 3000, green = 2000, orange= 1000.

The study population comprised of 104 patients undergoing IMRT after MRM for breast cancer at the Second Affiliated Hospital of Dalian Medical University from 2017.1 to 2021.12. Women aged 18 years or older with stages II and III were eligible for our study. All patients underwent unilateral mastectomy and PMRT, with or without immediate IBBR, and were confirmed as breast cancer by surgical pathology. Only patients with new primary breast cancers were included. Radiotherapy was performed on the ipsilateral chest wall, supraclavicular and infraclavicular lymph nodes at a dose of 50Gy/2Gy/25f. The target area was delineated referring to the target mapping of the Radiation Therapy Oncology Group (RTOG) (**Figure 1**). Exclusion criteria included recurrence or bilateral breast cancer, serious dysfunction of the heart, brain, liver, and kidney, other malignant tumors, or history of radiation to the chest before mastectomy. The patients were divided into two groups according to whether undergoing breast reconstruction. In the first group, all patients underwent immediate implant-based breast reconstruction instead of autologous breast reconstruction after modified radical mastectomy. Patients in the second group received modified radical mastectomy without breast reconstrution. The first group was named IBBR group with 46 patients. The second group was named non-reconstruction group with 58 patients. This study was approved by the Ethics Committee of the Second Affiliated Hospital of Dalian Medical University. All patients were aware of this study and signed informed consent.

Clinicopathological data and radiotherapy dosimetric parameters of all enrolled patients were collected. Clinicopathological data included age at diagnosis, body mass index (BMI), laterality, clinical stage, axillary lymph node metastasis, pathological type, tumor location, and histological grade. Radiotherapy dosimetric parameters included: the volume, mean dose (Dmean), near-minimal dose (D98), near-maximal dose (D2) of planning target volume (PTV), PTV receiving 95% of the prescription dose (V95), PTV receiving 105% of prescription dose (V105), Dmean to the ipsilateral lung, the volume percentages of ipsilateral lung receiving more than 5Gy, 10Gy, 20Gy, 30Gy and 40 Gy (V5, V10, V20, V30 and V40), Dmean to the bilateral lung, the volume percentages of bilateral lung receiving more than 5Gy, 20Gy (V5, V20), the volume percentages of contralateral breast receiving more than 1Gy, 5Gy (V1, V5), and so on. Acute toxicity radiation, such as radiation pneumonia (RP) and radiation dermatitis (RD), was defined as the maximum toxicity both during therapy and 6 weeks after completion. RP and RD assessments were reported corresponding to the RTOG acute radiation injury grading criteria (16). We collected FACT-B scale questionnaire from patients 6 months after radiotherapy to investigate their quality of life. We also followed up the implant-related complications up to 6 months after radiation therapy, such as wound infection, skin necrosis, capsular contracture, implant rupture, or exposure. Clinicopathological data, complications of radiotherapy, and functional assessment of cancer therapy-breast cancer (FACT-B) scale between two groups were compared by chi-square test. The differences in radiotherapy dosimetric parameters were examined by independent samples t-test. All tests were two-sided and differences were considered statistically significant at P-value <0.05. Statistical analyses were carried out by SPSS25.0 software.

## 3 Results

### 3.1 Comparison of clinicopathological data

Compared to the non-reconstruction group, the IBBR group had more younger patients (<40 = 58.7% *vs*. 8.6%, P<0.05), earlier stage (II=56.5% *vs*. 32.8%, P<0.05) and fewer axillary lymph node metastases (<3 = 58.7% *vs*. 44.8%, P<0.05). BMI, laterality, pathological type, tumor location, and histological grade between the two groups were not statistically significantly different (see **Table 1**).

**Table 1:** Comparison of clinicopathologic data between the IBBR group and the Non-reconstruction group.

Clinicopathological data	IBBR(n=46)	Non-reconstruction(n=58)	*x* ^2^	P value
**Age(years)**			30.198	<0.001
<40	27 (58.7%)	5 (8.6%)		
≥40	19 (41.3%)	53 (91.4%)		
**BMI (Kg/m^2^)**			0.093	0.761
<24	24 (52.2%)	32 (55.2%)		
≥24	22 (47.8%)	26 (44.8%)		
**Laterality**			0.156	0.693
Left	24 (52.2%)	28 (48.3%)		
right	22 (47.8%)	30 (51.7%)		
**Clinical stage**			5.901	0.015
II	26 (56.5%)	19 (32.8%)		
III	20 (43.5%)	39 (67.2%)		
**Lymph nodes(N)**				
≤3	27 (58.7%)	26 (44.8%)	10.724	0.005
4-9	16 (37.8%)	13 (2.4%)		
≥10	3 (6.5%)	19 (32.8%)		
**Pathological type**			1.462	0.441
Invasive lobular carcinoma	3 (6.5%)	5 (8.6%)		
Invasive ductal carcinoma	41 (89.1%)	47 (81.0%)		
others	2 (4.3%)	6 (10.3%)		
**Tumor location**			1.741	0.419
Outer quadrant	35 (76.1%)	45 (77.6%)		
Inner quadrant	10 (21.7%)	9 (15.5%)		
Central region	1 (2.2%)	4 (6.9%)		
**Histological grade**			3.430	0.180
1-2	33 (71.7%)	33 (56.9%)		
3	10 (21.7%)	15 (25.9%)		
Unknown	3 (6.5%)	10 (17.2%)		

### 3.2 Dosimetric comparison of PTV and organs at risk

When analyzing the radiotherapy dosimetry of PTV between the two groups (**Table 2**), the results showed that the volume of PTV in the IBBR group was significantly larger than that in the non-reconstruction group (930.78cm^3^ *vs*.709.79cm^3^, P<0.05). Moreover, the Dmean and D2 of PTV in the IBBR group were slightly smaller than those in the non-reconstruction group, while the D98 and V95 of PTV were slightly larger than that in the non-reconstruction group. The above differences were all statistically significant (P<0.05). There was no significant difference in V105 of PTV between the two groups.

**Table 2:** Dosimetric comparison of PTV, lung and breast between the IBBR group and the Non- reconstruction group.

Planning objective	IBBR(n=46)	Non-reconstruction(n=58)	t	P value
PTV				
Volume (cm^3^)	930.78±255.65	709.79±215.96	4.777	<0.001
Dmean(Gy)	52.37±0.44	52.80±0.95	-3.293	0.001
D98 (Gy)	48.83±0.45	48.48±0.91	2.506	0.014
D2 (Gy)	55.12±1.44	56.62±3.00	-3.355	0.001
V95(%)	99.04±0.45	98.77±0.79	2.147	0.034
V105(%)	46.78±14.40	51.32±18.97	-1.347	0.181
Ipsilateral lung				
Dmean(Gy)	13.39±1.96	14.90±2.34	-3.497	0.001
V5(%)	47.90±9.12	51.81±9.60	-2.114	0.037
V10(%)	35.78±6.30	39.76±6.50	-3.145	0.002
V20(%)	25.07±4.81	28.32±5.32	-3.224	0.002
V30(%)	19.17±4.60	21.67±4.83	-2.677	0.009
V40(%)	13.66±4.29	16.42±4.48	-3.172	0.002
Bilateral lung				
Dmean(Gy)	7.23±1.99	7.89±1.45	-1.956	0.053
V5(%)	25.32±7.79	27.16±7.12	-1.254	0.213
V20(%)	12.81±3.54	14.10±3.10	-1.981	0.050
Contralateral breast				
V1(%)	37.84±23.01	40.26±20.1	-0.572	0.569
V5(%)	13.68±23.01	17.90±12.31	-1.548	0.125

When comparing the radiotherapy dosimetry of organs at risk (**Tables 2**, **3**), we found that the Dmean, V5, V10, V20, V30, and V40 of ipsilateral lung in the IBBR group were significantly lower than those in the non-reconstruction group (P<0.05). There was no significant difference in Dmean, V5, V20 of bilateral lung and V1, V5 of contralateral breast between the two groups (P > 0.05). Among patients with left breast cancer in our study (24 in the IBBR group and 28 in the non-reconstruction group), the Dmean, V10, V20, V30, and V40 of the heart in the IBBR group were all significantly lower than those in the non-reconstruction group, but there was no significant difference in V5 of heart between the two groups.

**Table 3:** Dosimetric comparison of heart in patients with left breast cancer between the IBBR group and the Non-reconstruction group.

Heart	IBBR(n=24)	Non-reconstruction(n=28)	t	P value
Dmean(Gy)	5.94±3.05	8.42±3.56	-2.683	0.010
V5(%)	25.40±17.82	34.64±17.86	-1.862	0.069
V10(%)	16.15±11.53	23.15±11.59	-2.217	0.031
V20(%)	8.12±6.06	13.44±6.58	-3.013	0.004
V30(%)	5.17±4.73	9.45±5.36	-3.027	0.004
V40(%)	3.29±3.59	6.77±4.36	-3.109	0.003

### 3.3 Complications of radiotherapy

#### 3.3.1 Acute toxicity of radiotherapy (RP and RD)

The occurrence of RP and RD in the two groups was shown in **Table 4**. The incidence of grade 1 RP was observed to be 17.4% and 20.7% in the two groups, respectively, while the incidence of grade 2 RP was found to be 4.3% and 5.2%, respectively. No cases of grade 3-4 RP were reported. In terms of RD, the incidence rates for grade 1 were recorded as 36.9% and 41.4% in the two groups, while for grade 2 they were noted as 15.3% and 12%, similarly, the incidences of grade 3 RD were documented as 8.7% and 6.9%, respectively. No instances of grade 4 RD were observed. There was no significant difference between the two groups (P>0.05). All patients in both groups successfully completed radiotherapy after appropriate management of adverse reactions.

**Table 4:** Comparison of RP and RD between the IBBR group and the Non-reconstruction group.

RP/RD	IBBR(n=46)	Non-reconstruction(n=58)	*x* ^2^	P value
**RP**			0.239	0.887
0	36 (78.3%)	43 (74.1%)		
1	8 (17.4%)	12 (20.7%)		
2	2 (4.3%)	3 (5.2%)		
**RD**			0.425	0.935
0	18 (39.1%)	23 (39.7%)		
1	17 (36.9%)	24 (41.4%)		
2	7 (15.3%)	7 (12.0%)		
3	4 (8.7%)	4 (6.9%)		

#### 3.3.2 Implant-related complications

During our follow-up, grade1-2 capsular contracture occurred in 8 patients in the IBBR group (17.4%), wound infection occurred in 5 patients (11.0%), and skin necrosis occurred in 3 patients(6.5%). No implant rupture or exposure occurred. All patients were treated for wound infection and skin necrosis prior to radiation therapy and all completed radiation therapy. The overall incidence rate was 34.9%. However, implant loss occurred in two patients after completion of radiotherapy due to infection, and the reconstruction failure rate was 4.3%.

### 3.4 Quality of life comparison

Quality of life in both groups was assessed 6 months after radiotherapy using the FACT-B scale (**Table 5**). The results showed that the scores of physiological status, social and family status, emotional status, functional status and additional attention in the IBBR group were significantly higher than those in the no reconstruction group (P<0.05).

**Table 5:** Quality of life comparison between the IBBR group and the Non-reconstruction group.

Quality of life	IBBR(n=46)	Non-reconstruction(n=58)	t	P value
Physiological status	12.30±3.18	9.76±2.22	4.609	<0.05
Social and family status	19.22±2.57	16.93±3.30	3.861	<0.05
Emotional status	16.46±2.46	13.22±2.90	6.04	<0.05
Functional status	18.43±3.26	13.59±2.70	8.298	<0.05
Additional attention	24.59±2.43	21.83±2.52	5.635	<0.05

## 4 Discussion

Compared with Western countries, traditional Chinese women have lower requirements for body image, especially for the elderly (11), which explains why more patients under 40 years old underwent breast reconstruction in this study. In addition, consistent with our results, a study consisting of 112348 patients from the Surveillance, Epidemiology, and End Results(SEER) database who underwent mastectomy showed that IBR was less popular in patients with later stage (17). This may be due to the surgeons considering PMRT for the patients with later stages before treatment. Many surgeons believe that PMRT is a relative contraindication of IBR because it can increase immediate breast reconstruction complications and reduce patient satisfaction (18, 19), especially for implant-based reconstruction. However, some studies indicated that with the progress of radiotherapy technology, the presence of breast reconstruction did not compromise the technical delivery of PMRT (20).

Motwani SB reported that radiation treatment planning after IBR was compromised because of the steeply sloped medial and apical contours of the reconstructed breast mound and the greater distance from the skin surface to the deep chest wall structures (21). Our study showed radiation treatment planning was better achieved in target dose coverage and dose distribution under the background of the popularization of IMRT. In addition, the effect of radiotherapy on organs at risk after IBR was controversial. For example, Jethwa KR and Liljegren A considered radiotherapy after IBR had no significant impact on ipsilateral lung and heart dose compared with the non-reconstruction group (22, 23). However, OHRI N suggested that breast reconstruction had a certain protective effect on organs at risk (20). We found that IBBR after modified radical mastectomy has a certain dose advantage for organs at risk, especially for the ipsilateral lung and heart in patients with left breast cancer. For all the patients in our study, the prosthesis was placed between the pectoralis major muscle and the pectoralis minor muscle, and the posterior chest wall CTV border extended beyond the posterior boundary of the pectoralis major muscle, only the anterior part of the prosthesis included. So the placement of the prosthesis increased the distance between the posterior chest wall CTV border and the ribs, contributing to better radiotherapy target dose distribution and lower cardiac and lung doses.

In the current study, no statistically significant difference in the incidence of RP was observed, 21.7% in the IBBR group and 25.9% in the non-reconstruction group respectively. Most RP were mild and moderate. In a study carried out by MangesiusJ including 396 breast cancer patients, the total incidence of RP was 38%, of which 10% were symptomatic (> grade 1) (24). The improved dose homogeneity achieved with IMRT may have explained the low RP rates in our study. In addition, although bolus was used in all patients in our study, the incidence of RD (> grade 1) was still lower than that reported by Jinli Ma (25), and there was no statistical difference between the two groups. This may be due to the application of skin protective agents during radiotherapy. In a systematic review, the incidence of implant-related complications was 41.3% and the rate of reconstruction failure was 16.8% in patients treated with IBBR combined with PMRT (26). The incidence of implant-related complications in this study was 34.9%, and the reconstruction failure rate was 4.3%, and the low incidence may be related to the short follow-up period. Additionally, we generally believed that radiotherapy was associated with higher implant-related complications in the past (27). However, Stuart SR compared the effect of radiation therapy on implant-related complications in breast cancer patients undergoing IBBR, 23.9% of patients who received PMRT had acute complications, 5.4% had late complications, 30.9% of non-irradiated patients had acute complications and 2.4% had late complications. Radiation therapy resulted in a higher, but not statistically significant risk of late complications compared to non-irradiated patients (28). It had also been suggested that age over 50 years and a larger mastectomy weight were associated with a higher risk of acute complications, but not with PMRT (29). It was evident that the relationship between radiotherapy and high implant-related complications is not absolute.

IBR can avoid the impact of breast defects on patients and improve their quality of life (15, 30), but radiotherapy may increase the complications of implant-based reconstruction, offsetting the benefits of breast reconstruction in terms of quality of life. For example, a study by the Mastectomy Reconstruction Outcomes Consortium (MROC) suggested that breast satisfaction was significantly lower in patients who received implant reconstruction and radiation therapy than in patients who received implant reconstruction without radiation therapy (31). Similarly, a prospective study by Chris et al. suggested that patients who underwent implant reconstruction and radiotherapy had significantly lower quality of life than those who did not undergo radiotherapy. In addition, breast satisfaction significantly worsened in patients undergoing implant reconstruction and radiation therapy compared with baseline values (32). Postmastectomy radiotherapy was associated with worse patient-reported outcomes after breast reconstruction. Despite these findings, it had also been shown that the need for reconstruction, patient self-assessed cosmetic outcomes and acceptability remain high despite the fact that radiotherapy may increase implant-related complications (33, 34), suggesting that women were willing to accept the potential risks of IBBR in exchange for its benefits. We used the FACT-B scale to evaluate the quality of life in patients with postoperative radiotherapy for breast cancer. The scores of physiological status, social and family status, emotional status, functional status, and additional attention in the IBBR group were higher than those in the non-reconstruction group. It was suggested that IBBR can improve the quality of life of patients after modified radical breast cancer surgery even if adjuvant radiotherapy was performed.

## 5 Conclusion

Patients younger than 40 years old or at stage II are more likely to receive immediate breast reconstruction after modified radical mastectomy. With the advancement of radiotherapy technology, patients with IBBR can achieve better radiation dosimetry distribution and higher quality of life without more complications of radiotherapy. In a word, IBBR is a reasonable option for patients who need radiotherapy after modified radical mastectomy.

## Data availability statement

The original contributions presented in the study are included in the article/supplementary material. Further inquiries can be directed to the corresponding authors.

## Ethics statement

The studies involving humans were approved by The Ethics Committee of the Second Affiliated Hospital of Dalian Medical University. The studies were conducted in accordance with the local legislation and institutional requirements. The participants provided their written informed consent to participate in this study.

## Author contributions

All authors contributed to the review conception and design. The first draft of the manuscript was written by YZ and all authors commented on previous versions of the manuscript. All authors contributed to the article and approved the submitted version.
